# Myricetin ameliorates atherosclerosis in the low-density-lipoprotein receptor knockout mice by suppression of cholesterol accumulation in macrophage foam cells

**DOI:** 10.1186/s12986-019-0354-7

**Published:** 2019-04-25

**Authors:** Zhe Meng, Mengyu Wang, Junhui Xing, Yuzhou Liu, Haiyu Li

**Affiliations:** Department of Cardiology, The First Affiliated Hospital of Zhengzhou University, Zhengzhou, 450052 Henan China

**Keywords:** Myricetin, Macrophage, Atherosclerosis, CD36

## Abstract

**Background:**

Myricetin, a major flavonoid found in several foods including berries, grapes and wine, exhibited strong antioxidant potency, yet the effect on atherosclerosis is not fully understood. In this study, we examined the effect of myricetin on lipid accumulation in macrophage and atherosclerosis in atherosclerosis-prone low density lipoprotein receptor-deficient (*Ldlr*^*−/−*^) mice.

**Methods:**

*Ldlr*^*−/−*^ mice were fed an atherogenic diet supplemented with myricetin (0.15% in the diet, *v*/v) for 8 weeks. Body weight, adipose tissue weight, food intake, serum biochemical parameters were measured. Atherosclerosis lesions and macrophages accumulaton in lesions were analyzed and quantified. Macrophages were exposed to 20 μM of myricetin before incubated with oxidized low-density lipoprotein (ox-LDL) (25μg/mL) or Dil-ox-LDL for the indicated time. Lipid uptake and foam cell formation were evaluated by flow cytometry and microscopy. The intracellular lipids were extracted and measured. mRNA expression and protein of cholesterol metabolism related receptors were analyzed.

**Results:**

Myricetin administration reduced the weight, plasma lipid levels but not food intake in *Ldlr*^*−/−*^ mice when fed an atherogenic diet. Myceritin-treated *Ldlr*^*−/−*^ mice displayed significantly less atherosclerotic areas and macrophages in the cross sections of the aortic root. There were also less lipophilic areas in *En face* Oil red O staining of aorta from myceritin-treated *Ldlr*^*−/−*^ mice. Myceritin treatment also markedly ameliorated ox-LDL-induced cholesterol accumulation in macrophages. The expression of CD36 were decreased in myricetin treated macrophages with ox-LDL incubation, while scavenger receptors class A (SR-A) and scavenger receptors class B (SR-BI) expression was not altered, indicating that these effect of myricetin were dependent on CD36 pathway.

**Conclusions:**

Our findings indicated that myricetin suppressed cholesterol accumulation in macrophage foam cells by inhibition of CD36-mediated ox-LDL uptake, and suggested myricetin may have an important therapeutic function for atherosclerosis.

## Background

Atherosclerosis, a leading causes of death worldwide, is characterized by excessive cholesterol deposition within the artery wall resulting in myocardiac infarction and stroke [1, 2] . Atherosclerosis is a chronic inflammatory disorder of the vascular wall involving of many circulating immune cells, such as monocytes, lymphocytes, and plateles [3]. Particularly, infiltration of monocytes and their subsequent formation of macrophage-derived foam cells and proinflammatory cytokine secretion is a crucial step in the progression of atherosclerosis [4, 5]. Modified LDL such as ox-LDL causes excess cholesterol deposition in macrophages and leads to the formation of foam cells that play a crucial role in the initiation and progression of atherosclerosis [6, 7].

Myricetin is a naturally flavonoid extracted from wine, berries, grapes, vegetables and medicinal herbs. Flavonoids exhibited anti-inflammatory activity and had been reported as potent natural antioxidants that protect against modified LDL uptake in macrophages and attenuated the development of atherosclerosis [8, 9]. The beneficial effect of red wine consumption against the development of atherosclerosis may help to explain the ‘French Paradox’- a low incidence of cardiovascular events in southern France despite a diet rich in saturated fat [10, 11]. Myricetin has been shown to possess anti-inflammation and antioxidation properties [12, 13]. And, myricetin has also demonstrated the ability to anti-obesity, improve glucose utilization, modulate lipid metabolism [14–16]. It was reported that myricetinreduced the weight-gain, feed efficiency, level of blood lipids, adipocyte size, and weight and size of the perirenal and epididymal adipose tissues [16]. This anti-obesity effect may be involved in upregulation of adropin and β-endorphin levels. Furthermore, myricetin has been found to reduce hyperglycemia, ameliorating the impaired insulin-signaling pathway and improve glucose utilization in diabetes-like animal models [14, 17]. However, the effect of myceritin on the lipid metabolism and atherosclerosis is not fully understood.

We explored the interaction between myricetin effects and atherosclerosis used atherosclerosis-prone *Ldlr*^*−/−*^ mice. *Ldlr*^*−/−*^ mice fed an atherogenic diet (AsD) displayed dislipidemia and accelerated atherosclerosis. This study investigated the effects of myricetin on lipid metabolism and atherosclerosis in AsD fed *Ldlr*^*−/−*^ mice. In addition to its anti-obesity effect, myricetin has beneficial effects on dislipidemia and atherosclerosis. The possible mechanism by which myricetin ameliorating atherosclerosis may be involved in improved lipid metabolism and suppression of cholesterol accumulation in macrophages.

## Methods

### Animals

*Ldlr*^*−/−*^ mice were purchased from Jackson Laboratories (Bar Harbor, ME). All experiments involving mice were approved by the Institutional Animal Care Research Advisory Committee of the National Institute of Biological Science (NIBS) and Animal Care Committee of Zhengzhou University. *Ldlr*^*−/−*^ mice were divided into 2 groups: Control group (*n* = 10), Myr group (*n* = 10). Control group were given an AsD (20% fat and 0.5% cholesterol). Myr group were fed with AsD and supplemented with myricetin (0.15% in the diet, *v*/v). All mice were maintained on a 12:12-h light-dark cycle and have free access to water and food. Body weight and food intake were monitored throughout the experiments.

### Cell culture

Peritoneal macrophages were isolated from WT mice 3 days after intraperitoneal injection with a 4% solution of thioglycollate media. Isolated peritoneal macrophages were cultured in DMEM supplemented with 10% fetal bovine serum. Peritoneal macrophages were plated on chamber slides in 12-well plates. Treatments including serum starvation (DMEM only), myricetin treatment ((20 μmol L^− 1^) or control vehicle (DMSO).

### Blood metabolite analysis

Blood was obtained by retro-orbital bleeding. Plasma total cholesterol (TC), and triglyceride (TG) were determined by enzymatic methods (Sigma kits, USA).

### Atherosclerosis lesion analysis

12-week-old *Ldlr*^*−/−*^ mice were fed an AsD (20% fat and 0.5% cholesterol). The aorta and aortic sinus sections were prepared and stained as described [18]. The lesion area of each aorta and aortic sinus were analyzed by using Image J software. Sections of aortic sinus were subjected to immunohistochemical staining with MAC-2 antibody (Santa Cruz Biotechnology, Dallas, Texas) for macrophages.

### Lipid uptake assay

For Dil-oxLDL uptake macrophages was serum-starved in DMEM for 24 h. Macrophages were treated with or without myricetin (20 μmol L^− 1^) for 20 h, then 10 g/mL Dil-oxLDL at 4 °C for 4 h. Cells were washed and lysates were analyzed by fluorometry (Molecular Devices, Downingtown, PA) with 514 nm excitation and 550 nm emission.

### Foam cell formation

Macrophages were cultured on chamber slides. Cells were starved for 24 h and treated with myricetin ((20 μmol L^− 1^) for 12 h, then cells were incubated with ox-LDL (25μg/mL) for another 12 h. Upon fixation with PFA (4%) cells were stained with Oil-red O and evaluated by microscopy. To evaluate cholesterol ester accumulation, cells were incubated with BODIPHY 493/503(Invitrogen) for the indicated time and flow cytometric analysis was performed. After 24 h incubation with ox-LDL, cells were washed 3 times with PBS, and then free cholesterol (FC), cholesterol ester (CE) and triglyceride (TG) were determined using commercial kits from Applygen Technologies (Beijing, China).

### Western blot analysis

For Western blotting, cells were lysed using RIPA buffer. Equal amounts of protein (30 μg) were loaded and separated by 8% SDS-PAGE. Separated proteins were electrophoretically transferred to a nitrocellulose membrane, blocked with 5% (w v^− 1^) skim milk solution for 1 h, and then incubated with primary antibodies to CD36 (Santa Cruz Biotechnology, Inc., CA, USA; Cat. No. sc-9154), SR-B1 (Abcam, Inc.; Cat. No. ab-106,572), ABCA1 (Abcam, Inc.; Cat. No. ab-18,180) and GAPDH (Abcam, Inc.; Cat. No. ab-181,602), respectively, overnight at 4 °C. Blots were visualized by an ECL system (Pierce).

### RNA isolation and quantitative real-time PCR

Total RNA was extracted using Trizol reagent (Invitrogen, USA) and first-strand cDNA was generated by using an RT kit (Invitrogen,USA). Amplifications were performed using an opticon continuous fluorescence detection system (MJ Research) with SYBR green fluorescence (Molecular Probes, Eugene, USA). All samples were quantitated by using the comparative CT method for relative quantitation of gene expression, normalized to GAPDH. The following primers were used: CD36 (Forward: 5 -GGC AGG AGT GCT GGA TTA-3; Reverse: 5 -GAG GCG GGC ATA GTA TCA-3); SR-A (Forward: 5 -TTA AAG GTG ATC GGG GAC AAA-3; Reverse: 5 -CAA CCA GTC GAA CTG TCT TAA G-3; SR-BI (Forward: 5 -AAC ACG TAC CTC CCA GAC ATG CTT-3; Reverse: 5 -AGT CGT CCA TTG CCA CAG-3); ABCA1(Forward: 5-CCC AGA GCA AAA AGC GAC TC-3; Reverse: 5-GGT CAT CAT CAC TTT GGT CCT TG-3); IL-10 (Forward: 5 -GAC CAG CTG GAC AAC ATA CTG CTA A-3; Reverse: 5 -GAT AAG GCT TGG CAA CCC AAG TAA-3); IL-6 (Forward: 5 -AGG CTC CGA GAT GAA CAA-3; Reverse: 5 -AAG GCA TTA GAA ACA GTC C-3); MCP-1 (Forward: 5 -TCC CAA TGA GTA GGC TGG AG-3; Reverse: 5 -AAG TGC TTG AGG TGG TTG TG-3); GAPDH (Forward: 5 -TGA TGA CAT CAA GAA GGT GGT GAA G-3; Reverse: 5 -TCC TTG GAG GCC ATG TAG GCC AT-3).

### Statistical analysis

All data are presented as means ± SD. SPSS 19.0 was used to perform statistical analysis of the data. The Shapiro-Wilk test was performed to determine the distribution of the variables and non-normal distributions were log-transformed before statistical analysis with an independent t-Test. Variables that were analyzed with Mann-Whitney U test. A value of *P* < 0.05 was considered statistically significant.

## Results

### Myricetin ameliorates metabolic abnormalities in low density lipoprotein receptor-deficient (*Ldlr*^*−/−*^) mice fed an atherogenic diet

**Fig. 1:**
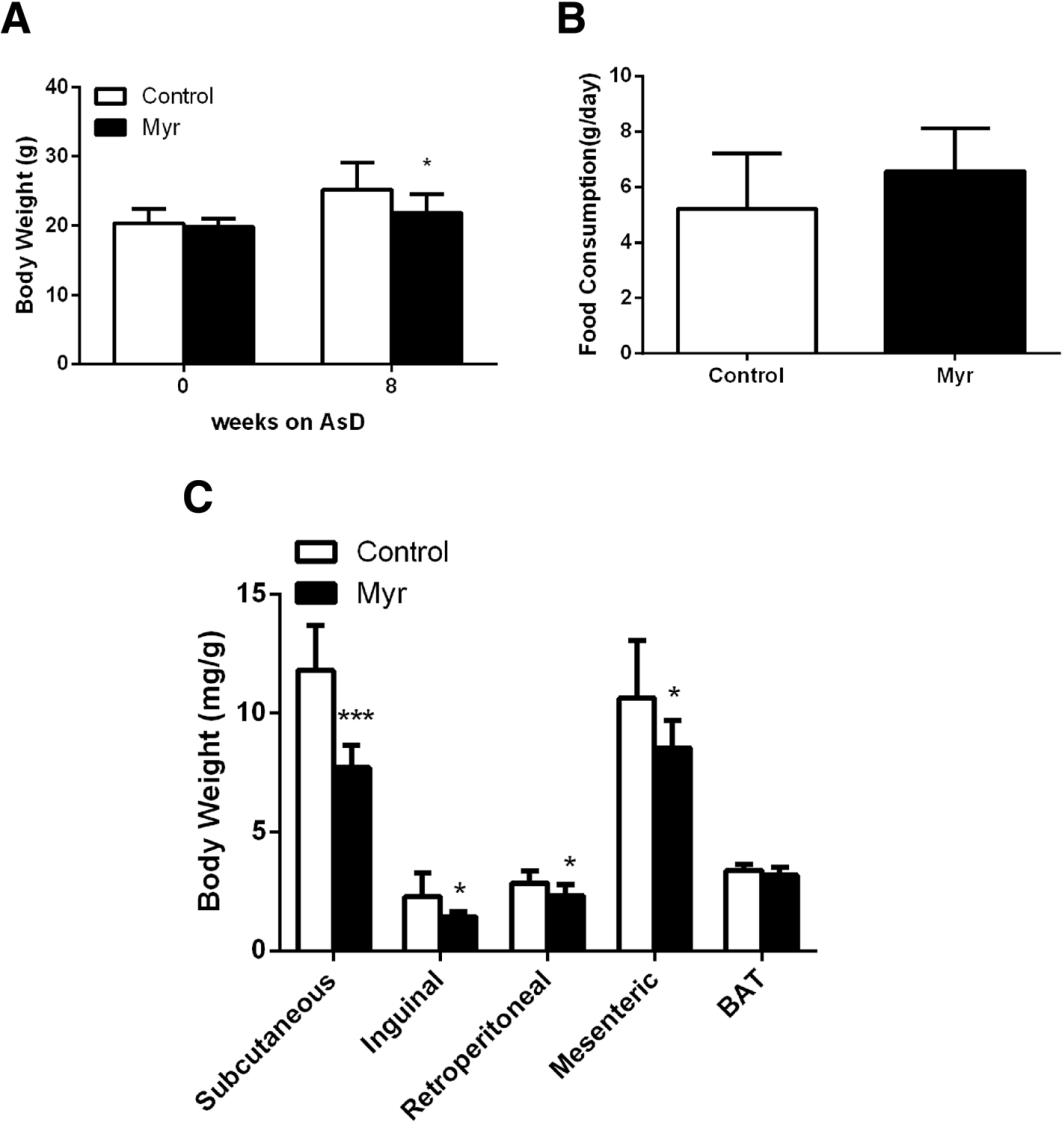
Effect of myricetin on body weight and food intake. Body weight (**a**), Food intake (**b**) and Weights of fat pads (**c**) in Control mice and Myr mice. Values are expressed as mean ± SD. *N* = 10, *P < 0.05, ***P < 0.001 for Myr mice vs. Control mice

As shown in Fig. 1a, the body weight of myricetin-treated mice was significantly lower than that of mice in the Control group (21.86 ± 0.90 g vs 25.22 ± 1.30 g,*P*<0.05) after feed with AsD for 8 weeks. No significantly difference in the total food intake was observed between Control group and Myr group mice (*P* = 0.19, Fig. 1b). As shown in Fig. 1c, the subcutaneous white adipose tissue weight (WAT) of Myr group mice was 7.72 ± 0.30 mg, this value was lower than that of Control group, 11.81 ± 0.63 mg, *P*<0.001. The inguinal WAT, retroperitoneal WAT, and mesenteric WAT weight of Myr group mice were 1.45 ± 0.07 mg, 2.32 ± 0.16 mg and 8.55 ± 0.38 mg, respectively. These values were lower than that of Control group, 2.29 ± 0.33 mg, 2.86 ± 0.33 mg and 10.63 ± 0.86 mg, respectively, *P*<0.05.

**Fig. 2:**
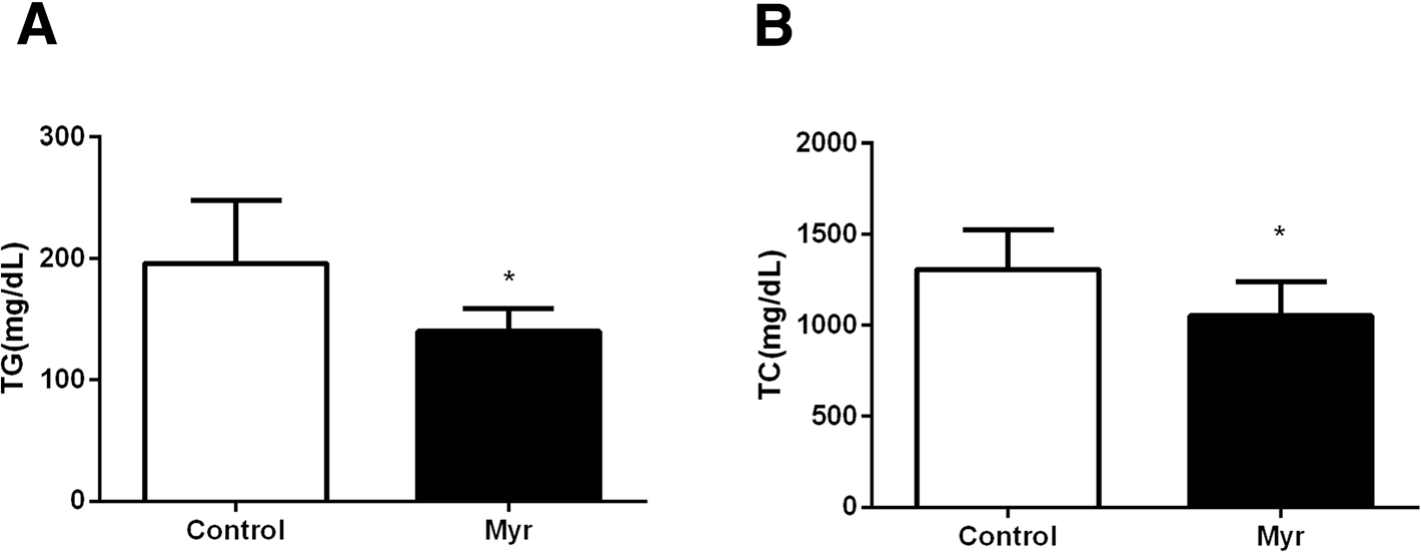
Effect on plasma lipid. Plasma TG level (**a**) and Plasma TC level (**b**) in Control mice and Myr mice. Values are expressed as mean ± SD. *N* = 10, *P < 0.05, for Myr mice vs. Control mice

Plasma lipid levels were then analyzed. As shown in Fig. 2, the AsD caused the elevation of plasma TC, TG concentrations in mice. The TG and TC levels in Myr group mice, 140.1 ± 18.46 mg/dL and 1052 ± 60.16 mg/dL, were significantly lower (*P*<0.05) than those of the Control group mice, 196.3 ± 17.28 mg/dL, 1308 ± 68.63 mg/dL. Taken together, these results show that myricetin can reduce body weighr and lipid accumulation without decreasing food intake in AsD fed *Ldlr*^*−/−*^ mice.

### Myricetin alleviates atherosclerosis and macrophage accumulation in atherosclerotic lesions

**Fig. 3:**
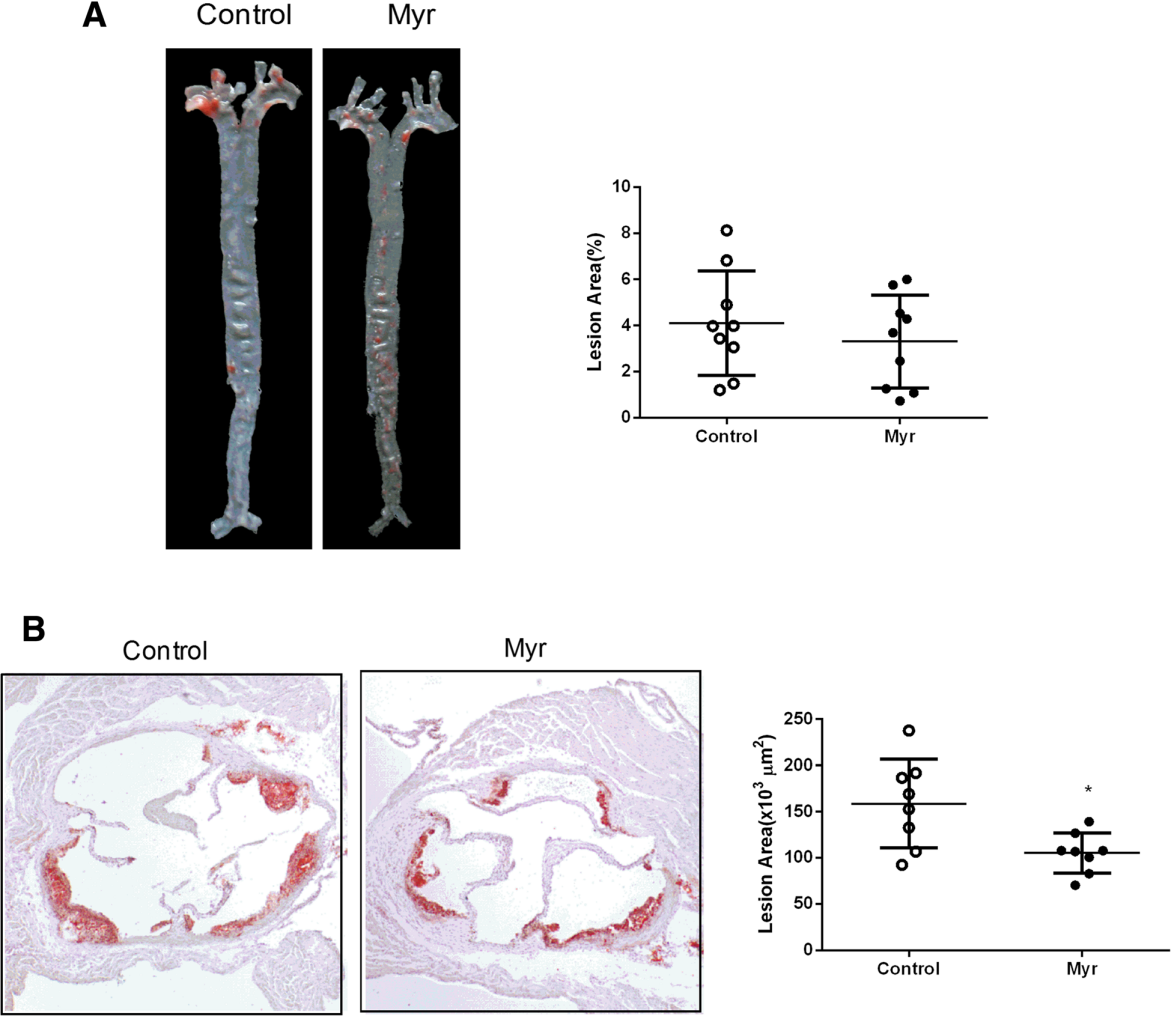
Effect on atherosclerosis lesions. Representative *en face* images of oil red O-stained aorta and quantification of lesion area (**a**) and Representative aortic root sections stained with oil red O and quantification of aortic lesion areas (**b**). Values are expressed as mean ± SD. *N* = 10, *P < 0.05, for Myr mice vs. Control mice

To investigate whether myricetin affects the formation of atherosclerotic lesions, *Ldlr*^*−/−*^ mice were placed on AsD with or without myricetin administration for 8 weeks. After 8 weeks, most lesions developed in ascending arota and aortic arch. We performed en face analysis of the aorta and the results showed Myr group mice developed less atherosclerotic lesions compared to Control mice, however, the difference did not reach statistical significance (Fig. 3a) (3.31 ± 0.67% vs 4.111 ± 0.75%, *P* = 0.44). Quantification of Oil red-stained cross sections of aortic roots showed reduced plaque size in Myr mice when compared with Control mice (Fig. 3b) (105.2 ± 7.77% × 10^3^ μm^2^ vs 158.8 ± 16.94 × 10^3^ μm^2^, *P*<0.05). These results indicate that myricetin may have a protective role in atherosclerosis.

**Fig. 4:**
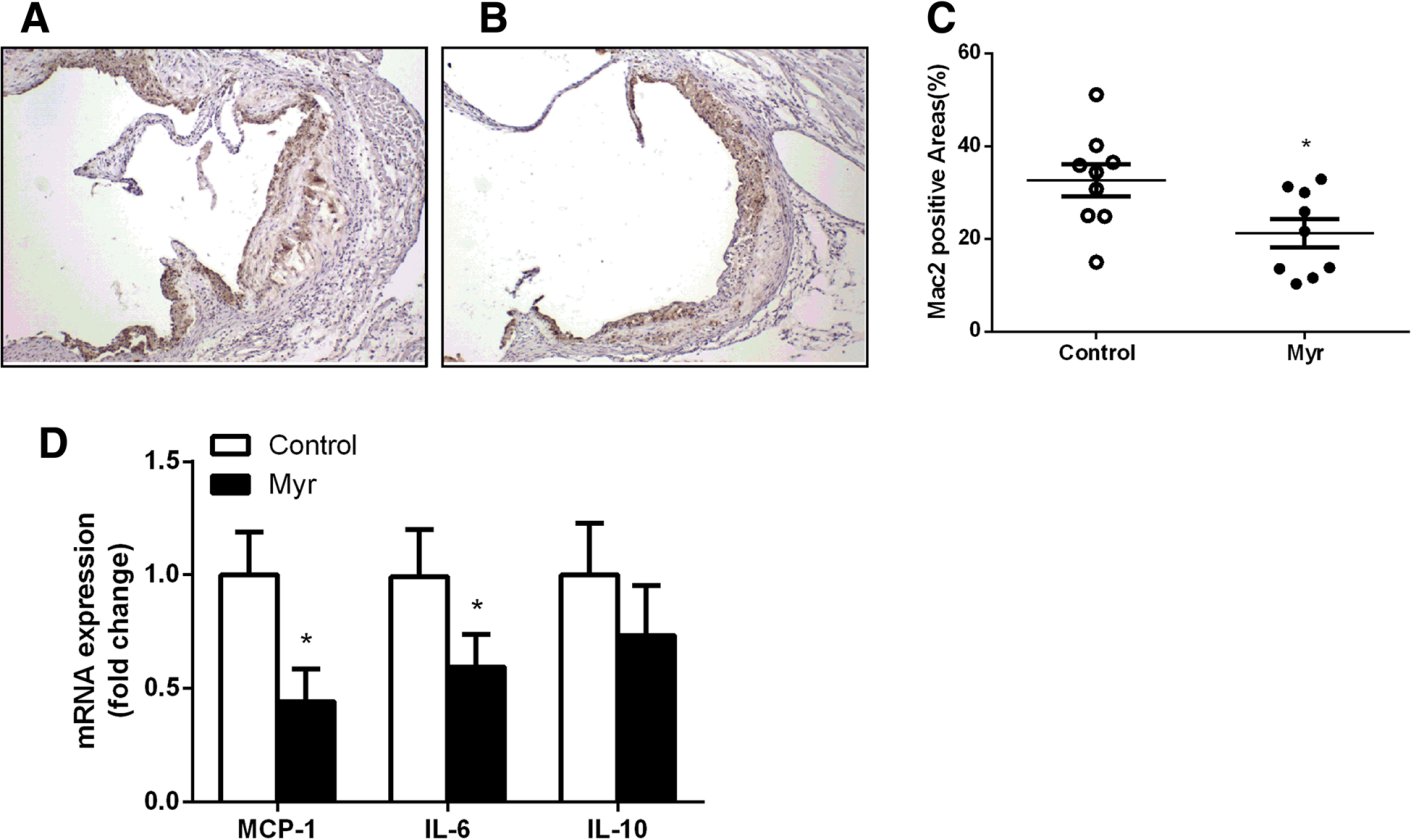
Effect on macrophage accumulation in the atherosclerotic lesions. Quantification of macrophages infiltration in the aortic sinus lesions stained with anti-Mac2 antisera in Control mice (**a**) and Myr mice (**b**) after 8 weeks on AsD. **c** Comparison of the percentage of Mac2-positive areas in the aortic sinus lesion from Control mice and Myr mice. **d** RT-PCR analysis of MCP-1, IL-6 and IL-10 mRNA expression in atherosclerosis lesionfrom Control mice and Myr mice. Values are expressed as mean ± SD. *N* = 9, *P < 0.05, for Myr mice vs. Control mice

To evaluate whether myricetin affects macrophage accumulation in the atherosclerotic lesions, we performed immunohistochemical staining of the aortic root tissue sections with an antibody that recognizes murine macrophages, Mac-2. The Mac-2-positive areas were analyzed with NIH Image J. There were more Mac-2-positive cells in the aortic lesions of Control mice when compared with Myr mice (Fig. 4a and b). Mac-2 positive area: 32.67 ± 3.46% vs 21.22 ± 3.03%, *P*<0.05. However, there was little or no difference in the amount of smooth muscle cells and collagen content within plaque lesions (data not shown). Furthermore, the expression of pro-inflammatory mediators such as MCP-1 and IL-6 were also reduced in the aorta of Myr mice (Fig. 4d). The expression of IL-10 in Myr mice was not different compared with Control mice (Fig. 4d). These findings indicate that myricetin treatment is not only important in regulating plaque size, but also, in determining plaque composition and morphology, which may have potential impact on plaque stability.

### Myricetin regulates foam cell formation

**Fig. 5:**
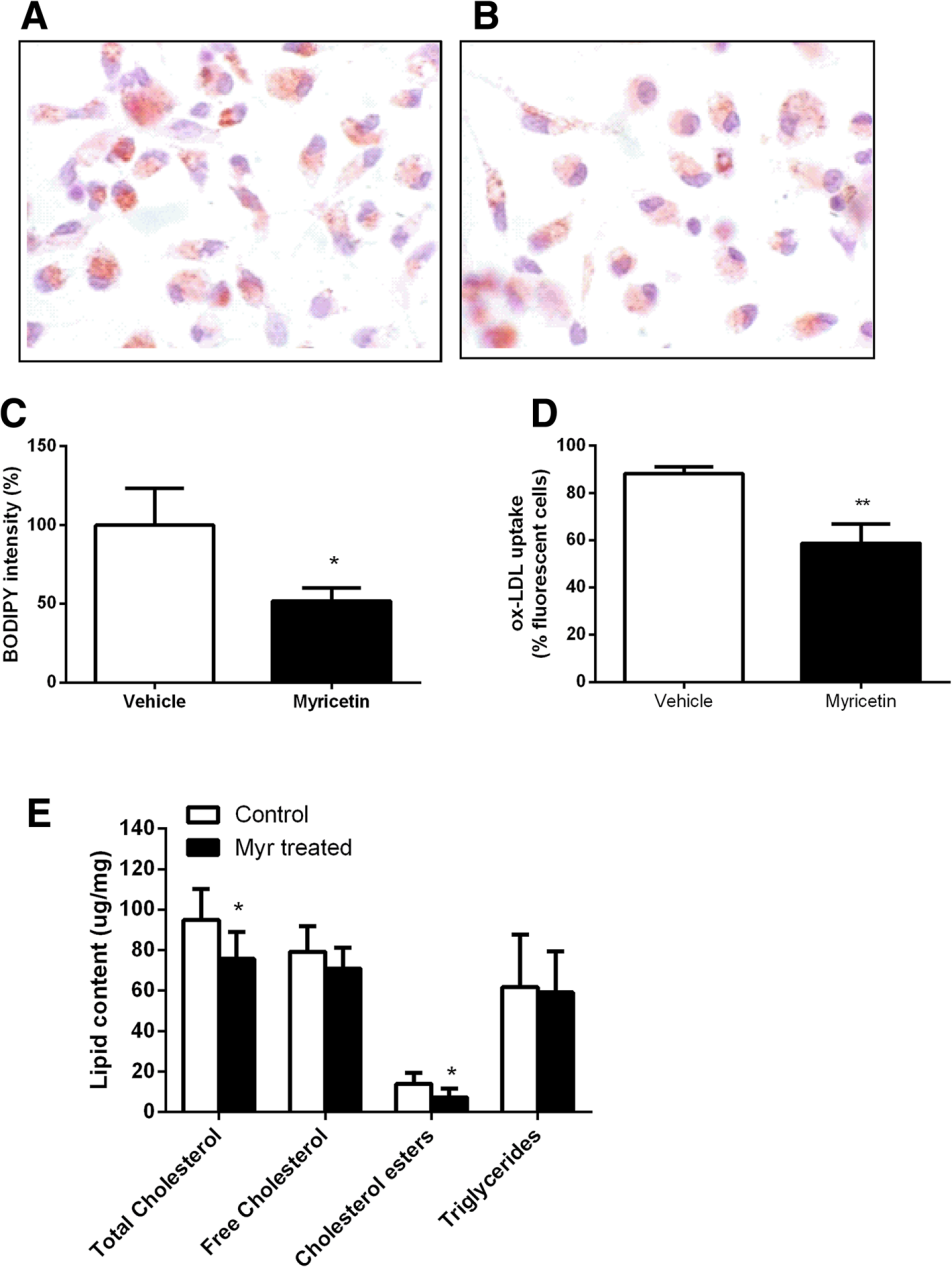
Effect on foam cell formation. Foam cell formation with vehicle (**a**) and myricetin (**b**) treated macrophages with ox-LDLincubation. ox-LDL uptake (**c**) and BODIPY intensity (**d**) by flow-cytometry in cholesterol load macrophages. **e** Lipid accumulation in vehicle and myricetin treated macrophages. Values are expressed as mean ± SD. *P < 0.05,**P < 0.005 for Myricetin vs. Vehicle

To evaluate whether myricetin has a functional role in regulating lipid uptake, we thus explored the effect of myricetin on ox-LDL-induced foam cell formation. Treatment with myricetin markedly decreased ox-LDL induced cholesterol accumulation in macrophages, as revealed by cellular cholesterol content and Oil-red O staining (Fig. 5a, b and e) (75.70 ± 5.43 μg/mg vs 94.74 ± 6.28 μg/mg, *P*<0.05). Therefore, myricetin may regulate cholesterol metabolism of macrophage foam cells.

Consistent with decreased foam cell formation, BODIPY fluoresence, which is a marker of cellular lipid droplets, was reduced in macrophages treated with myricetin (Fig. 5c). Furthermore, cholesterol ester content was decreased in macrophages upon treatment with ox-LDL as compared to vehicle macrophages (Fig. 5e) (7.288 ± 1.80 μg/mg vs 13.90 ± 2.23 μg/mg, *P*<0.05). However, Myricetin did not alter the intracellular triglyceride accumulation induced by ox-LDL (Fig. 5e) (59.03 ± 8.23 μg/mg vs 61.63 ± 11.57 μg/mg, *P*<0.05).. Taken together, these findings suggest that myricetin plays a critical role in macrophage foam cell formation.

### The mechanisms of reduced lipid accumulation in myricetin treated macrophages

**Fig. 6:**
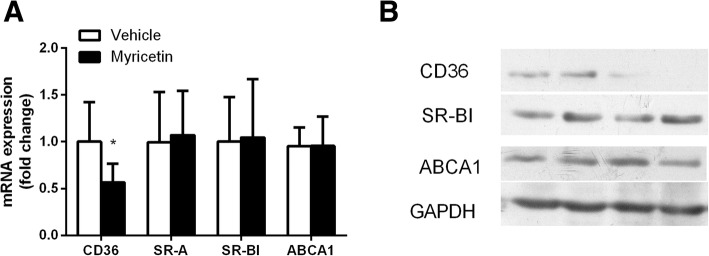
The Mechanisms of reduced lipid accumulation in myricetin treated macrophages. mRNA (**a**) and protein (**b**) expression of CD36, scavenger receptor-A (SR-A), scavenger receptor- BI (SR-BI) and ATP-binding cassette transporter A1 (ABCA1) in macrophages stimulated with ox-LDL. Values are expressed as mean ± SD. *P < 0.05, for Myricetin vs. Vehicle

To explore the mechanism underlying the protective effect of myricetin, we examined the expression of several key factors in atherogenesis in macrophages. The expression of CD36 mRNA were decreased in myricetin treated macrophages with ox-LDL incubation (*P*<0.05), while SR-A and SR-BI expression was not altered (Fig. 6a). Immunoblotting results showed that the protein level of CD36 was significantly reduced in myricetin treated macrophages (Fig. 6b).

Upon cholesterol loading, ABCA-1 mRNA and protein levels were not altered in vehicle and myricetin treated macrophages (Fig. 6a and 6b).

## Discussion

The main purpose of this study was to evaluate the effect of myricetin on atherosclerotic development using *Ldlr*^*−/−*^ mice. Myricetin is a narurally occuring flavonoid and the anti-inflammation effect of myricetin has well been established [19, 20]. However, the efficacy of myricetin on atherogenesis and the mechanism ramained elusive. The present study demonstrates that myricetin has an inhibitory effect on the development of atherosclerotic lesions in atherosclerosis-prone *Ldlr*^*−/−*^ mice, which could be related to suppression of cholesterol accumulation in macrophage foam cells. In macrophages, incubation with myricetin attenuated ox-LDL-induced cholesterol accumulation, and thiseffect may be involved in reduction of CD36-dependent ox-LDL uptake. Our findings strongly indicate that myricetin has a protective effect in maintenance of cholesterol homeostasis during the formation of foam cells in atherogenesis.

Myricetin naturally exists in many foods, especially in wine and grapes. Recent studys have demonstrated many health benefits of myricetin including antioxidative [21], anticarcinogenic [22], antiplatelet activities [10], cytoprotective effects [23], anti-obesity [16], improving glucose utilization [17] and plasma lipids profile [15]. In our study, myricetin treatment reduced body weight but not food intake, this anti-obesity effect is consistent with previous findings [16]. When *Ldlr*^*−/−*^ mice were fed an AsD for 8 weeks, plasma cholesterol reached as high as 1300 mg/dL. And, plasma cholesterol decreased significantly (~ 1000 mg/dL) after myricetin administration on the same diet. The mechanism of lowering plasma cholesterol may be related to increasing the fatty acid oxidation mediating via peroxisome proliferatior-activated receptor alpha (PPARα) activation which was reported in a previous study [15]. As high plasma triglyceride and cholesterol level increases the risk of atherosclerosis, the beneficial effect of myricetin for hypercholesterolemia and hypertriglyceridemia may caused profound reductions in atherosclerosis. The anti-atherogenesis effect of myricetin were shown in Oil red O staining of *En face* and the aortic root*.* Strikingly, histological analysis of aortic root lesions revealed significantly less macrophages in atherosclerotic plaques of Myr mice when comparedwith Control mice, but no difference in the amount of smooth muscle cells and collagen content. Taken together, myricetin may have a protective role in atherosclerosis, not only ameliorating plaque size, but also maintaining plaque stability.

Macrophages play a crucial role in atherogenesis [6], and uptake of modified LDL by macrophage and then transformation into foam cells contribute to development and progression of atherosclerosis. The lipid homeostasis in macrophages depends on the balance of influx and efflux of lipid in which scavenger receptors (SRs) are critically involved [24–26]. At least two members of this family, CD36 and scavenger receptors class A (SR-A), mediate the internalization of ox-LDL by macrophages, leading to cholesterol ester accumulation and foam cell formation [27, 28]. In contrast, the efflux of intracellular cholesterol to high-density lipoprotein is mediated by reverse cholesterol transporters, including class B scavenger receptor type I (SR-BI) and ATP-binding cassette transporter A1 (ABCA1) [29–31]. Interestingly, the uptake of modified LDL was inhibited in myricetin-treated macrophages, which is consistent with downregulation of CD36 expression. In the present study, attenuation of CD36 by myricetin but not SR-A or SR-BI, contribute to decreased cholesterol accumulation in macrophages. Meanwhile, foam cell formation was decreased in myricetin-treated macrophages which can be partly attributed to myricetin effect on cholesterol esterification. Indeed, cholesterol ester formation was reduced in myricetin-treated macrophages upon ox-LDL loading, and cholesterol efflux was not affected. The mechanism of reduced cholesterol ester formation remains unclear.

The aim of this study was to investigate the anti-atherosclerotic activity of myricetin. The study was performed with *Ldlr*^*−/−*^ mice which are characterized by accelerated development of atherosclerosis along with hypercholesterolemia. Despite the current widespread use of *Ldlr*^*−/−*^ mice to mimic human atherosclerosis, obvious differences in murine genetic and metabolic profiles are observed [32, 33]. Hamsters display similar lipoprotein profiles and coronary atherosclerosis which could be an ideal animal for translational research of human atherosclerosis [34, 35]. Recently, *Ldlr*^*−/−*^ hamster were successfully generated and serve as an ideal platform over other small animal models for basis and translational research of atherosclerosis [36]. Convincing evidence should be provided in *Ldlr*^*−/−*^ hamster and we have plan to carry our this project. In summary, we have demonstrated that myricetin not only improved dyslipidemia, but also inhibit foam cell formation. These findings indicated that myricetin has therapeutic potentials in the prevention of atherogenic cardiovascular diseases.

## Conclusions

In conclusion, our results show that myricetin regulates cholesterol uptake in macrophages through CD-36 pathway, resulting in an attenuation of atherosclerosis lesions. This findings suggested myricetin may have an important therapeutic function for atherosclerosis.

## Abbreviations

ABCA1: ATP-binding cassette transporter A1
AsD: Atherogenic diet
*Ldlr*: Low-density-lipoprotein receptor
ox-LDL: Oxidized low-density lipoprotein
PPARα: Peroxisome proliferatior-activated receptor alpha
SR-A: Scavenger receptors class A
SR-BI: Scavenger receptors class B
SRs: Scavenger receptors
TC: Total cholesterol
TG: Triglyceride and HDL-cholesterol
WAT: White adipose tissue
